# Evaluation of human epididymis protein 4 (HE4) and Risk of Ovarian Malignancy Algorithm (ROMA) as diagnostic tools of type I and type II epithelial ovarian cancer in Japanese women

**DOI:** 10.1007/s13277-014-2738-7

**Published:** 2014-10-19

**Authors:** Hiroyuki Fujiwara, Mitsuaki Suzuki, Nobuhiro Takeshima, Ken Takizawa, Eizo Kimura, Toru Nakanishi, Kyosuke Yamada, Hirokuni Takano, Hiroshi Sasaki, Koji Koyama, Kazunori Ochiai

**Affiliations:** 1 Department of Obstetrics and Gynecology, Jichi Medical University, 3311-1 Yakushiji, Shimotsuke, Tochigi 329-0498 Japan; 2 Department of Gynecology, Cancer Institute Hospital, 3-8-31, Ariake, Koto-ku, Tokyo 135-8550 Japan; 3 Department of Gynecologic Oncology, Kosei General Hospital, 5-25-15, Yayoicho, Nakano-ku, Tokyo 164-8617 Japan; 4 Department of Gynecology, Aichi Cancer Center Hospital, 1-1 Kanokoden, Chikusa-ku, Nagoya, 464-8681 Japan; 5 Department of Gynecologic Oncology, The Jikei University School of Medicine, 3-25-8, Nishi-Shimbashi, Minato-ku, Tokyo 105-8461 Japan; 6 Department of Obstetrics and Gynecology, Jikei University Kashiwa Hospital, 163-1, Kashiwashita, Kashiwa, Chiba 277-8567 Japan; 7 Center for Preventive Medicine, OCAT Clinic, 1-4-1, Minatomachi, Naniwa-ku, Osaka, 556-0017 Japan

**Keywords:** HE4, ROMA, Epithelial ovarian cancer, CA125, Type I and type II EOC

## Abstract

Human epididymis protein 4 (HE4) levels and the Risk of Ovarian Malignancy Algorithm (ROMA) have recently been shown to improve the sensitivity and specificity of epithelial ovarian cancer (EOC) diagnosis. We evaluated HE4 levels and ROMA as diagnostic tools of type I and type II EOC in Japanese women. Women who had a pelvic mass on imaging and were scheduled to undergo surgery were enrolled as ovarian mass patients. Serum levels of carbohydrate antigen 125 (CA125) and HE4 were tested in 319 women (131 benign, 19 borderline, 75 malignant, and 94 healthy controls). CA125, HE4, and ROMA were evaluated for sensitivity and by receiver operating characteristics (ROC) in type I and type II EOC. The results showed that, at 75 % specificity, the sensitivity of CA125 and HE4 for type II was 92.1 % for both markers and for type I was 51.5 % and 78.8 %, respectively. The sensitivities of ROMA (type I, 84.8 % and type II, 97.4 %) were better than those of CA125 and HE4. CA125, HE4, and ROMA were all highly accurate markers for type II. For type I, HE4 and ROMA showed better sensitivity than CA125. ROMA displayed the best diagnostic power for type I and type II including for the early stage of type I. In conclusion, HE4, CA125, and ROMA are valuable markers for type II EOC diagnosis. HE4 and ROMA analyses may improve differentiation between type I EOC and a benign mass. Measurement of combined HE4 and CA125 levels provides a more accurate method for EOC diagnosis.

## Introduction

Ovarian cancer is the seventh most common cancer and the seventh cause of death from cancer in women worldwide and is the most common type of gynecological malignancy. In Japan, the incidence rate was 8.4 per 100,000 women in 2012 [1]. The symptoms of ovarian cancer are related to the presence of adnexal masses and are often vague and unspecific. The primary goal of the diagnostic evaluation of an adnexal mass is to determine whether it is benign or malignant. Ultrasound is used to assess patients for ovarian cancer and, while it is effective in detecting pelvic masses, it has a low specificity for determination of whether a mass is benign or malignant. Specificity is improved by using a Doppler ultrasound and a morphology index but performance varies among different operators [2, 3].

Epithelial ovarian cancer (EOC) histological subtypes have different outcomes and may require different treatments [4]. The four major histological subtypes are serous, endometrioid, clear cell, and mucinous. Recent morphological and molecular genetic studies have led to the development of a new paradigm for the pathogenesis and origin of EOC based on a dualistic model of carcinogenesis that divides EOC into two categories: type I and type II. Type I tumors are suggested to behave in an indolent manner, are more often confined to the ovary at diagnosis, have a stable genome, and do not have TP53 mutations, although somatic mutations are frequently detected in a number of genes [5]. Type II tumors are suggested to be more aggressive and are genetically highly unstable; the majority of type II tumors have TP53 mutations, and almost half of the cases have mutation, hyper-methylation, or dysfunction of BRCA1/2 [6]. These aggressive tumors account for 75 % of all EOC and are responsible for 90 % of deaths from the disease [5, 7].

Currently, carbohydrate antigen 125 (CA125) is the most widely used tumor marker for women with a pelvic mass suggestive of ovarian cancer. However, its predictive power is insufficient. It is elevated in about 80 % of women with EOC but only in 50 % of women with early stage disease [8]. The specificity of CA125 is limited, since it can also be elevated in a range of common benign conditions such as endometriosis and fibroids [9].

In recent years, the use of novel biomarkers such as human epididymis protein 4 (HE4) has been studied to improve the sensitivity and specificity of ovarian cancer diagnosis. HE4 is primarily expressed in the reproductive and respiratory tracts [10, 11] and is overexpressed in EOC [12]. The HE4 gene product is an *N*-glycosylated protein which is secreted into the extracellular environment and can be detected in the bloodstream of patients with ovarian cancer [13]. HE4 was found to be elevated in more than half of ovarian tumors that do not express CA125 [13]. This finding prompted the development of a dual marker algorithm that combined HE4 and CA125 with the pre- and postmenopausal statuses of the patient, known as Risk of Ovarian Malignancy Algorithm (ROMA) [14]. ROMA has been shown in several studies to better predict the presence of a malignant ovarian mass than other markers, with high sensitivity and specificity [14–18]. However, there is little data evaluating the use of HE4 and ROMA in a Japanese population.

We conducted a prospective multicenter study in Japan to evaluate the performance of HE4 and ROMA in predicting the risk of type I and type II EOC in Japanese women.

## Materials and methods

This was a prospective multicenter study involving seven study centers in Japan. Between 2012 and 2013, women between 20 and 79 years of age, who were diagnosed with an adnexal mass by ultrasound, CT scan, PET scan, or MRI, were enrolled as ovarian mass patients. Patients who were treated with neoadjuvant chemotherapy were excluded. Healthy controls were recruited from people who had a medical health check examination. Women without an adnexal mass by PET scan or MRI were enrolled as healthy controls (age 20–79). Women with previous bilateral oophorectomy, any gynecologic disease, or pregnancy were excluded. The study was approved by the institution's review board at each site and complied with the declaration of Helsinki. Written informed consent was obtained from all subjects. The study protocol was registered at the University Hospital Medical Information Network (UMIN) Clinical Trials Registry (protocol ID UMIN000006747).

Blood samples were collected after the pelvic mass was confirmed, and surgery was scheduled within 42 days between blood collection and surgery. The blood was drawn into a serum or serum separator tube, centrifuged, and frozen. The samples were stored at −20 °C or colder at the individual study sites and were shipped on dry ice to the laboratory at Abbott Japan (Matsudo, Japan). The specimens were thawed, aliquoted, and stored frozen at −70 °C until the analysis was carried out. After surgery, the tumors were examined by an experienced pathologist for diagnosis, histological analysis, grading, and staging (I–IV), according to the International Federation of Gynecologists and Obstetricians (FIGO) standards. The EOCs were then further classified into type I and type II tumors. Type I included low-grade (G1) serous, G1 endometrioid, all clear cell, mucinous, and transitional (Brenner) carcinomas. Type II included high-grade (G2–G3) serous, G2–G3 endometrioid, and malignant mixed mesodermal tumors.

The samples were tested at the Abbott Japan laboratory, using the ARCHITECT CA125 II, ARCHITECT HE4, ARCHITECT FSH, ARCHITECT Estradiol, and ARCHITECT Progesterone assays (Abbott Diagnostics, Abbott Park, IL) according to the manufacturer's instructions. If the menopausal status was not available from the medical chart, the women's age, follicle stimulating hormone (FSH), and estradiol values were used to assign menopausal status. We categorized women who did not have menopausal status in the medical chart into the postmenopausal group in cases where the woman was older than 60 years of age or the FSH level was ≥22 mIU/mL and the estradiol level was <20 pg/mL.

ROMA was calculated as per the HE4 package insert: for premenopausal women: predictive index (PI) = −12.0 + 2.38 × LN[HE4] + 0.0626 × LN[CA125] and for postmenopausal women: PI = −8.09 + 1.04 × LN[HE4] + 0.732 × LN[CA125], where LN = natural log. ROMA was calculated from the PI as follows: ROMA = exp(PI)/[1 + exp(PI)] × 100.

The sensitivity, specificity, positive predictive value (PPV), and negative predictive value (NPV) were calculated using the diagnosis of the individual hospitals. In addition, the sensitivity at 75 % specificity of patients with benign diseases was calculated since it was reported that the minimum sensitivity of ROMA that would be clinically useful would be when 75 % of patients with benign diseases were correctly classified as low-risk [15]. The cutoff values at 75 % specificity of patients with benign diseases are shown in Table 3. Receiver operating characteristic (ROC) plots were constructed and the area under the curve (AUC) was calculated for each marker and ROMA. Statistical differences in individual markers and ROMA between groups were evaluated using the Mann–Whitney *U* test or the Dunn test. Correlation between CA125 and HE4 was analyzed by using the Pearson correlation test. A *p* value of <0.05 was considered as statistically significant. Data were analyzed using Analyse-It version 2.22 (Analyse-It Software Ltd., Leeds, UK) for Microsoft Excel and JMP version 10.0.2 (SAS Institute Inc., North Carolina, USA).

## Results

### Patient characteristics

Between 2012 and 2013, 225 ovarian mass patients (131 benign, 19 borderline pelvic mass, and 75 malignant mass) and 94 healthy controls were enrolled. Four tumors were excluded because of non-epithelial ovarian cancer. Table 1 shows the background of participants. The EOCs were divided into the slow-growing type I EOC and the more aggressive type II EOC based on histology and grade (Table 2).

**Table 1:** Age, menopause status, and tumor status of the patients

		Mean age (range)	Total number	
Healthy control	Pre-M	32 (22–48)	46	
	Post-M	62 (22–48)	48	
	All (%)	48	94	(29.5 %)
Benign	Pre-M	36 (20–55)	92	
	Post-M	62 (41–79)	39	
	All (%)	43	131	(41.1 %)
Borderline	Pre-M	35 (25–47)	10	
	Post-M	62 (51–72)	9	
	All (%)	47	19	(6.0 %)
Malignant	Pre-M	43 (23–54)	33	
	Post-M	62 (49–77)	42	
	All (%)	54	75	(23.5 %)

*Pre-M* premenopausal, *Post-M* postmenopausal

**Table 2:** Type I and Type II classification of the epithelial ovarian cancers

Histology	Stage	Total	Histology	Stage	Total	
Mucinous	I	5	Mixed	I		
	III	4				
	Total	9 (12.7 %)		Total	3 (4.2 %)	
		Type I			Type II	
Clear cell	I	17				
	III	3				
	IV	1				
	Total	21 (29.6 %)				
		Type I				
Histology	Stage	Grade				Total
		G1	G2	G2–3	G3	
Serous	I	1			1	2
	II		1		2	3
	III	2	6	1	15	24
	IV				3	3
	Total	3	7	1	21	32 (45.1 %)
		Type I	Type II	Type II	Type II	
Endometrioid	I		2			2
	II		2			2
	III				2	2
	Total		4		2	6 (8.5 %)
			Type II	Type II	Type II	
Total		33 (46.5 %)		38 (53.5 %)		
		Type I		Type II		

### Sensitivity and AUC of CA125, HE4, and ROMA in type I and type II EOC

CA125, HE4, and ROMA values determined in pre- and postmenopausal women with benign diseases or with type I or type II EOC are shown in Table 3. HE4 and CA125 values significantly separated (*p* < 0.001) the type I and type II EOCs from the benign diseases (Table 3). The sensitivity of CA125 and HE4 at 75 % specificity of patients with benign diseases for type II was 92.1 % for both markers and was 51.5 % and 78.8 %, respectively, for type I (Table 3). The sensitivity of HE4 for type I EOC was better than that of CA125. Also, the sensitivities of ROMA for type I and II EOC (type I, 84.8 % and type II, 97.4 %) were better than those of CA125 and HE4 (Table 3).

**Table 3:** Statistical differences between HE4, CA125, and ROMA values and sensitivity, PPV, NPV, and AUC analyses, according to menopausal status and tumor status and histological stage

	Benign		Type I EOC						Type II EOC					
	Median	Cutoff value (75 percentile)	Median	*p* value^a^	ROC AUC	Sensitivity^b^ (%)	PPV (%)	NPV (%)	Median	*p* value^a^	ROC AUC	Sensitivity^b^ (%)	PPV (%)	NPV (%)
CA125 (U/mL)														
Total	21.9	57.0	61.2	<0.001	0.76	51.5	34.0	86.0	567.2	<0.001	0.92	92.1	51.5	97.0
Pre-M	23.3	62.1	145.6	0.001	0.77	56.3	28.1	90.8	383.4	<0.001	0.94	92.9	36.1	98.6
Post-M	11.3	31.5	56.8	<0.001	0.81	64.7	52.4	82.9	701.9	<0.001	0.92	95.8	69.7	96.7
Early stage			42.0	0.002	0.70	39.1	21.4	87.5	186.2	0.001	0.81	72.7	19.5	97.0
Late stage			179.6	<0.001	0.88	80.0	19.5	98.0	721.7	<0.001	0.96	100.0	45.0	100.0
HE4 (pmol/L)														
Total	40.8	48.5	65.8	<0.001	0.82	78.8	44.1	93.3	310.9	<0.001	0.95	92.1	51.5	97.0
Pre-M	39.2	44.0	63.5	<0.001	0.84	81.3	36.1	95.8	135.4	<0.001	0.99	100.0	37.8	100.0
Post-M	48.4	63.3	96.8	0.003	0.75	58.8	50.0	80.6	502.1	<0.001	0.91	87.5	67.7	90.6
Early stage			55.6	<0.001	0.75	69.6	32.7	93.3	92.6	<0.001	0.87	72.7	19.5	97.0
Late stage			171.8	<0.001	0.99	100.0	23.3	100.0	478.1	<0.001	0.99	100.0	45.0	100.0
ROMA (%)														
Total	5.6 %	8.8 %	24.8 %	<0.001	0.85	84.8	45.9	95.1	92.4 %	<0.001	0.96	97.4	52.9	99.0
Pre-M	4.4 %	6.0 %	12.8 %	<0.001	0.84	75.0	34.3	94.5	61.7 %	<0.001	0.99	100.0	37.8	100.0
Post-M	11.0 %	19.6 %	40.1 %	<0.001	0.83	70.6	54.5	85.3	98.3 %	<0.001	0.93	91.7	68.8	93.5
Early stage			13.5 %	<0.001	0.80	78.3	35.3	95.1	42.0 %	<0.001	0.92	90.9	23.3	99.0
Late stage			61.8 %	<0.001	0.98	100.0	23.3	100.0	97.8 %	<0.001	0.98	100.0	45.0	100.0

*Pre-M* premenopausal, *Post-M* postmenopausal

^a^The *p* value was calculated as a statistical difference compared to benign diseases by Mann–Whitney *U* test

^b^The sensitivity was calculated at 75 % specificity of patients with benign diseases

**Fig. 1:**
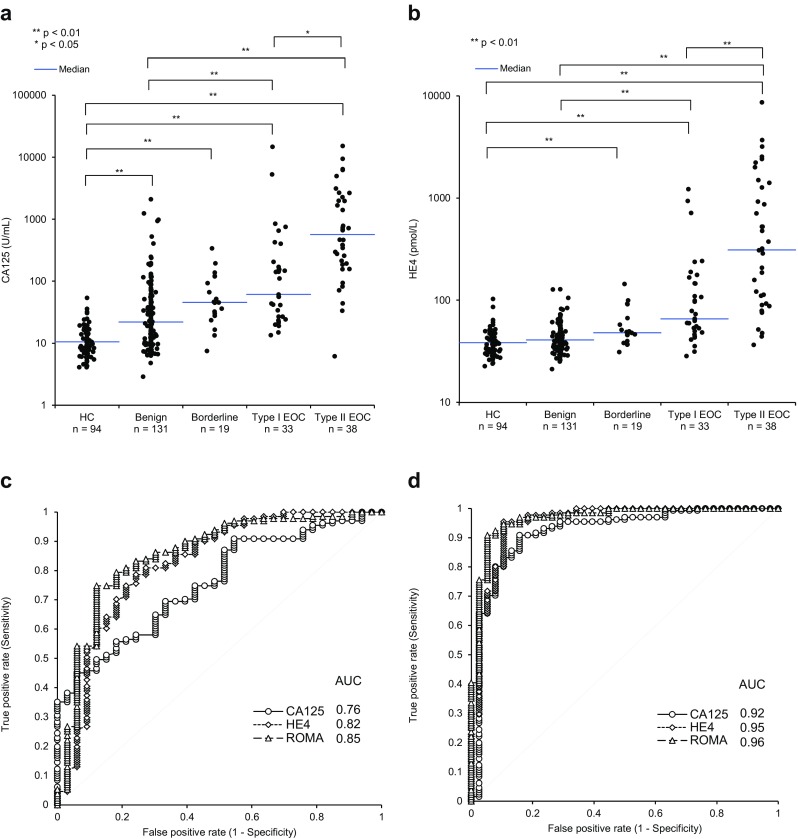
Scatterplot analysis of the correlation between CA125 and HE4 with tumor status, and ROC analysis of CA125, HE4, and ROMA between benign and EOC types. Scatterplots of **a** CA125 and **b** HE4 levels in healthy controls, benign and borderline tumor, and in type I and type II EOC; ROC analysis and AUC calculation for CA125, HE4, and ROMA in a comparison of **c** benign and type I EOC and **d** benign and type II EOC. The *p* value of the statistical differences between groups was calculated using the Dunn test

The levels of CA125 and HE4 in patients with type I and type II EOC were significantly higher than those in healthy controls and in patients with benign diseases. The median (range) and standard deviation in healthy controls of CA125 were 10.5 U/mL (4.1–54.2 U/mL) and 7.5 U/mL, respectively, and those of HE4 were 38.4 pmol/L (22.7–102.9 pmol/L) and 11.7 pmol/L, respectively. The level of HE4 in patients with benign diseases was equivalent to that of healthy controls, whereas the level of CA125 in patients with benign diseases was higher than that of healthy controls (Fig. 1a, b). The AUC was high for all markers when benign tumors were compared to type II EOC (0.92 CA125; 0.95 HE4; 0.96 ROMA), while the AUC was lower (0.76 CA125; 0.82 HE4; 0.85 ROMA) when benign tumors were compared to type I EOC (Fig. 1c, d).

### Evaluation of HE4, CA125, and ROMA in early and late stage type I and type II EOC

**Fig. 2:**
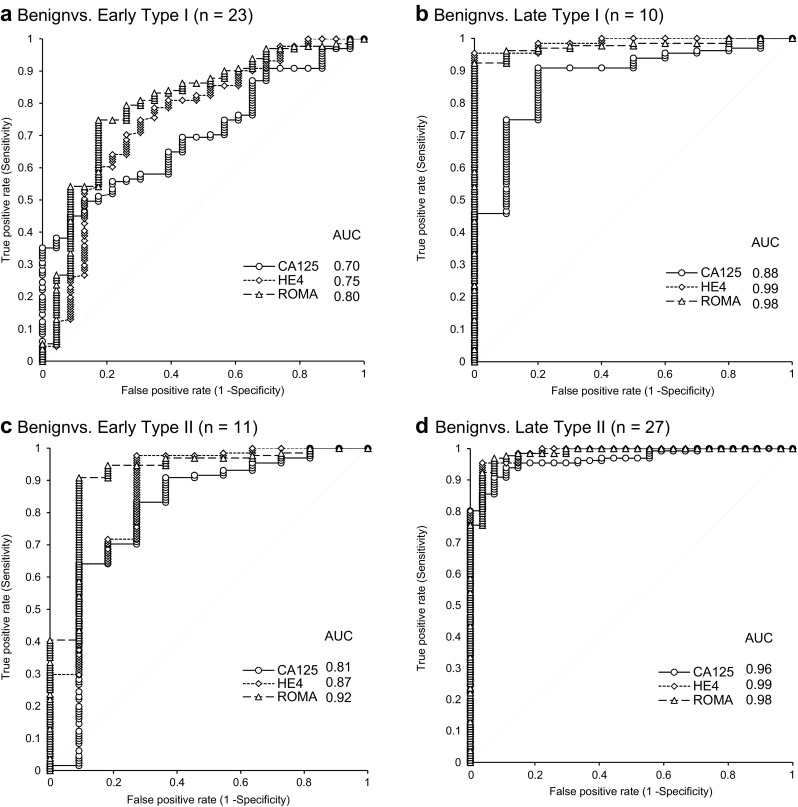
ROC analysis and AUC calculation for CA125, HE4, and ROMA in a comparison of benign and EOC tumors of different type. ROC analysis and AUC calculation for CA125, HE4, and ROMA in a comparison of **a** benign and early type I EOC, **b** benign and late type I EOC, **c** benign and early type II EOC, and **d** benign and late type II EOC

We next evaluated the AUC and sensitivity of HE4, CA125, and ROMA values according to FIGO tumor stages. Type I was divided into early (FIGO I + II; *n* = 23) and late (FIGO III + IV; *n* = 10) stages, and type II was also divided into early (*n* = 11) and late (*n* = 27) stages, which were compared to benign diseases. HE4 and CA125 levels significantly differentiated (*p* < 0.001) the early and late stage of both types of EOC from the benign diseases (Table 3). The AUCs for ROMA in late stage type I and type II EOC compared with benign diseases were 0.98 for both. The AUCs for ROMA in early stage type I and type II EOC compared with benign diseases were 0.8 and 0.92, respectively (Fig. 2a–d).

### Evaluation of CA125 and HE4 levels according to EOC histological subtype

**Fig. 3:**
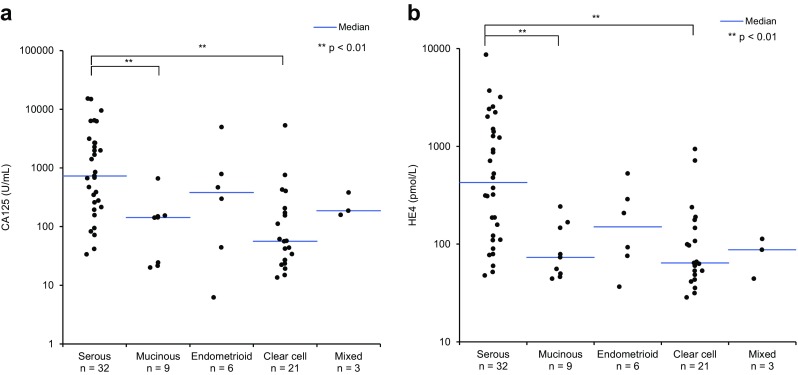
Correlation between CA125 and HE4 levels and tumor histology. Scatterplots of **a** CA125 and **b** HE4 levels in EOC tumors of different histology. The *p* value of the statistical differences between groups was calculated using the Dunn test

The division of EOC into the type I and type II EOC is based on evidence of genetic changes in EOC histological subgroups. Evaluation of CA125 and HE4 according to EOC histological subtypes was performed. Low median CA125 values were detected in mucinous carcinomas (143 U/mL) and in clear cell carcinomas (56 U/mL), while median CA125 values for endometrioid and serous carcinomas were higher (381 and 727 U/mL, respectively). The lowest median HE4 values were found in clear cell carcinomas (64 pmol/L), with higher values found in mucinous (73 pmol/L), mixed (88 pmol/L), and endometrioid (150 pmol/L) carcinomas, and the highest value was found in serous carcinomas (297 U/ml) (Fig. 3).

### Correlation between CA125 and HE4 levels

**Fig. 4:**
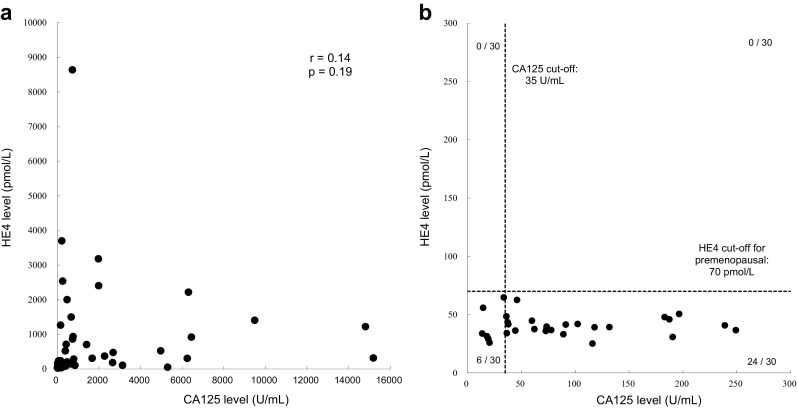
Correlation between CA125 and HE4 levels in EOC and in endometriotic cyst for premenopausal women. Scatterplots of serum levels of CA125 and HE4 in patients with EOC (**a**) and in patients with endometriotic cyst for premenopausal women (**b**). No significant relationship between CA125 and HE4 in patients with EOC was observed. The CA125 level was elevated to above cutoff value in 80 % (24/30) of the premenopausal patients with endometriotic cyst, whereas the HE4 level was not elevated

Scatterplots of serum levels of CA125 and HE4 in patients with EOC and in premenopausal patients with endometriotic cyst are shown in Fig. 4. The scatterplot was used to evaluate potential correlation between serum CA125 and HE4 levels in patients with EOC. However, no obvious linear trend was noted and the Pearson correlation coefficient was 0.14 (*p* = 0.19). When CA125 and HE4 levels in premenopausal patients with endometriotic cyst were evaluated, the CA125 level was elevated to above the cutoff value (35 U/mL) in 80 % (24/30) of cases, whereas the HE4 level was not elevated.

## Discussion

EOC is often detected at an advanced stage and is characterized by poor survival. As a result, it is a leading cause of death from gynecological malignancies. Improvement of the accuracy of diagnosis of EOC is therefore essential. It has recently been suggested that EOC can be subgrouped based on molecular genetic changes into a slow-growing type I EOC and an aggressive type II EOC. Tumors in the type I subgroup are generally larger in size and more often localized in the pelvis than the type II EOC. Therefore, the type I tumors are more easily detected at an earlier stage with conventional techniques than type II EOC [19]. It has been suggested that the aggressive type II EOC would benefit the most from detection at an early stage [20].

CA125 is currently the most widely used serum biomarker for detection of ovarian cancer. However, the major drawback of using CA125 as a single biomarker for EOC detection is its very low specificity. Thus, CA125 levels are also elevated in non-ovarian cancers (endometrial, endocervical, lung, and lymphoma) [21], in benign gynecological conditions (ovarian cyst, endometriosis, and myomas) [14, 21], and in some medical conditions (congestive heart failure and cirrhosis) [22]. Also, normal CA125 levels tend to be higher in premenopausal women, increasing the likelihood of false positive results when used in this group of women [14]. Moreover, CA125 levels may increase during pregnancy [23] and fluctuate throughout the menstrual cycle [24]. These observations cast several doubts on the possible impact of a “positive” result for CA125 when triaging women with signs or symptoms that may suggest an EOC. The search for new biomarkers with increased accuracy for EOC has led to identification of several molecules with a potential role in the diagnosis and triaging of adnexal masses. Of these markers, HE4 has been proposed to be highly relevant for such diagnosis. Moore et al. [14] proposed the use of ROMA for the differential diagnosis between benign and malignant lesions. Furthermore, they [25] showed that ROMA has a better diagnostic performance than the Risk of Malignancy Index. ROMA, which combines CA125 and HE4 values, yields a higher accuracy than either marker alone [13, 14]. However, there is little published information regarding the performance of HE4 and ROMA for the diagnosis of EOC type I and type II ovarian cancers.

We investigated the clinical utility of HE4 alone or of ROMA in assessing the likelihood of malignancy in Japanese women with a pelvic mass. This was a prospective clinical study that included women that were found to have a pelvic mass on ultrasound and were scheduled to undergo surgery, regardless of the menopausal status or the final pathology of the mass. The study population reflects real clinical situations where the use of tumor markers would be useful in further determining the nature of the mass.

In this study, the median serum levels of CA125 and HE4 were significantly higher in patients with type I and type II EOC than in patients with benign diseases and in healthy volunteers. However, the median serum level of CA125 in patients with benign diseases was significantly higher than that in healthy volunteers, whereas the median level of HE4 in patients with benign diseases was not significantly higher than that of healthy controls. In ROC analysis, the AUCs for ROMA and for HE4 alone were better than the AUC for CA125 in distinguishing between benign diseases and EOC. This result is consistent with the ROC analysis results reported by Chan et al. [26] and Sandri et al. [27]. These results suggest that HE4 and ROMA may be used as global universal tumor markers, since no ethnic differences were observed between Asian [26], Caucasian [27], and Japanese populations.

CA125, HE4, and ROMA were all highly accurate markers for type II EOC, with AUCs for type II EOC in comparison to benign diseases of greater than 0.92. On the other hand, the diagnostic abilities of CA125 and HE4 for early stage type I EOC were lower than that for type II EOC. These results are similar to those reported by Kristjansdottir et al. [28]. However, HE4 showed better sensitivity than CA125 (HE4, 78.8 %; CA125, 51.5 % at 75 % specificity of patients with benign diseases), when compared with benign diseases, which is due to the fact that the sensitivity of HE4 in premenopausal patients was better than that of CA125 in this study. CA125 levels in patients with endometriotic cyst were much higher than the HE4 levels. Although the ROMA results for type I and type II EOC were better than those of CA125 and HE4, even if ROMA was used, the sensitivity at 75 % specificity for early stage type I EOC (ROMA, 78.3 %) was insufficient. Improvement in the diagnostic ability to diagnose false negative results in early type I EOC is a challenge for the future.

Furthermore, the correlation between the levels of CA125 and HE4 in patients with EOC was poor. The combined data therefore suggest that HE4 either alone or in combination with CA125 is a valuable tumor marker for the diagnosis of EOC. Thus, measurement of the combination of HE4 and CA125 rather than measurement of either factor alone in a Japanese population provides a more accurate tool for the differential diagnosis of patients with EOC from those with benign diseases such as endometriotic cyst. A high serum HE4 level would suggest the presence of EOC, whereas elevated CA125 without elevated HE4 would suggest the presence of benign ovarian tumors or other benign diseases. Moreover, HE4 is a useful marker when the CA125 levels in patients are falsely elevated (e.g., during pregnancy).

This study has a limitation. We evaluated 319 samples in total; however, the number of samples with epithelial ovarian cancer was 75, with 19 more regarded as borderline. To reveal the usefulness of HE4 and ROMA more accurately, further evaluation of the value of these tools on a larger sample population, particularly from EOC patients, is needed.

In conclusion, CA125, HE4, and ROMA are valuable markers for type II EOC diagnosis. HE4 and ROMA analyses may improve the diagnostic ability for type I EOC compared to CA125 analysis alone. The measurement of the combination of HE4 and CA125 including ROMA analyses rather than measurement of either factor alone provides a more accurate method for the differential diagnosis of patients with EOC from those with benign diseases. However, the diagnostic power of CA125, HE4, and ROMA for the early stage of type I EOC might be lower than that for type II EOC. Therefore, discovery of early markers for the detection of type I EOC is a challenge for the future.
